# Water-soluble swab material for environmental sampling

**DOI:** 10.1128/aem.00030-26

**Published:** 2026-04-29

**Authors:** Kevin K. Crown, Timothy N. Lambert, Maria Kelly, Nelson S. Bell, Joshua Santarpia

**Affiliations:** 1 Department of WMD Threats & Aerosol Science, Sandia National Laboratories, Albuquerque, New Mexico, USA; 2 Department of Photovoltaics and Materials Technology, Sandia National Laboratories, Albuquerque, New Mexico, USA; 3 Sandia National Laboratories, Center for Integrated Nanotechnologies 1105, Albuquerque, New Mexico, USA; 4 Advanced Materials Laboratory, Sandia National Laboratories, Albuquerque, New Mexico, USA; 5 Global Center for Health Security and Department of Pathology, Microbiology and Immunology, University of Nebraska Medical Center, Omaha, Nebraska, USA; Centers for Disease Control and Prevention, Atlanta, Georgia, USA

**Keywords:** water-soluble swabs, polyvinyl alcohol (PVA) fibers, clearance sampling, environmental sampling, biological threat detection, *Bacillus thuringiensis *spores, Forcespinning technology, surface sampling efficiency, spore recovery, bioterrorism, contamination assessment, fiber characterization, cotton candy machine fiber production, microfluidic integration, environmental decontamination

## Abstract

Biological threats represent a growing vulnerability to national security, affecting both military and civilian populations. Deliberate dissemination of chemical and biological (CB) agents can have immediate and significant impacts on public health and the economy, highlighting the need for faster and more efficient techniques to assess contamination and safely clear affected areas for public reentry following an accidental or intentional biological release. Current surface sampling procedures set forth by the Centers for Disease Control and Prevention (CDC) call for sampling swab materials that are not soluble in aqueous bacterial growth medium. These have been shown here to be inefficient, releasing less than 40% of the captured biological material into aqueous solution for detection. Since the CDC put these procedures in place, there has been no impetus for industry to develop sampling swabs that release 100% of the sample they capture from a non-porous surface. This project developed cost-effective, novel, biologically hospitable, adsorbent materials that dissolve in water, releasing 100% of the captured sample. These fibers maintain structural integrity during sampling, can be premoistened without premature dissolution, and rapidly dissolve in solution, facilitating efficient sample release. Forcespun PVA fibrous swabs demonstrated significantly higher spore capture and release efficiency compared to traditional materials, without inhibiting bacterial growth or spore germination. These swabs could significantly improve current sampling technologies and procedures via increased spore detection and analysis efficiency. Higher sampling efficiency could translate to fewer samples required for analysis and significantly less time to render an area safe for public reentry. Increased sampling efficiency would also provide faster impact data to decision makers and lead to improved consequence management. Prior to this study, water-soluble swabs suitable for this purpose have not yet been developed.

**IMPORTANCE:**

Accurate environmental sampling is critical for detecting low‐level microbial contamination in both public health and biodefense settings. Our water‐soluble PVA swab material achieves up to 100% spore release from non-porous surfaces, more than double the recovery of traditional swabs, while being compatible with scalable, low‐cost fabrication methods. This new tool will enable more sensitive, faster, and more reliable surface monitoring across a wide range of environmental and diagnostic applications.

## INTRODUCTION

Biological threats, whether accidental or deliberate, pose significant risks to national security by impacting both military and civilian populations (1). Rapid detection and remediation are critical to mitigating the public health and economic consequences of biological agent releases (1). Current environmental surface sampling protocols recommended by the Centers for Disease Control and Prevention (CDC) rely on swab materials that are water-insoluble and inefficient, allowing the recovery of only a fraction of total analyte for analysis, according to Broadwater et al., and indeed releasing less than 40% of captured biological material into solution for analysis by our estimation (2–8). This inefficient recovery compromises the accuracy of contamination assessments and prolongs the time required to render areas safe for public access (2, 6, 7). Despite these limitations, there has been little impetus to develop more efficient sampling tools. Advances in surface sampling materials could improve detection sensitivity and sampling efficiency, ultimately reducing the number of samples required and decreasing the time needed for environmental decontamination following a biological release (6).

In this study, we report the development of polyvinyl alcohol (PVA)-based swabs that dissolve completely in aqueous solutions, releasing 100% of the captured biological material. PVA was chosen for its water solubility, biological inertness, and ability to form uniform fibers via Forcespinning technology (9–13). Additionally, we replicated fiber production using a cotton candy machine to demonstrate scalability and cost efficiency.

We evaluated the performance of PVA fibers against conventional swab materials—cotton, rayon, and foam—which are currently recommended in CDC sampling protocols (3, 5). Testing was performed using *Bacillus thuringiensis* spores, a low health-risk surrogate for *Bacillus anthracis*. This innovation offers potential improvements to current biological threat detection methods, supporting faster and more reliable contamination assessments while providing compatibility with automated sampling technologies.

## MATERIALS AND METHODS

### Modified Forcespinning machines

PVA fibers were forcespun from the solutions using a FibeRio Cyclone L-1000M Forcespinning system with a custom-made spinneret to accommodate four luer-lock orifices (14). The modified head provides a planar chamber of 4″ diameter and 1/8″ depth for deposition of polymer solution, with four equally spaced (90° orientation) channels to a commercial luer-lock fitting suitable for attachment of standard needles of desired gauge size. Luer locks are oriented in the plane of rotation (horizontal) to enable direct flow induced by centrifugal forces. Stainless steel dispensing tips (30- and 32-guage, Nordson EFD) were attached to the spinneret to change Forcespinning conditions in extrusion.

Fibers were also forcespun using a Carnival King cotton candy machine with an added rheostat to control the temperature of the spinneret which houses the PVA dissolved in water. The collection bowl was also modified to include vertically oriented metal slates to catch the dry, forcespun fibers. A system to deliver the polymer solution to the spinneret was also designed, consisting of a syringe pump responsible for dispensing polymer solution through a flexible tube routed to the spinneret (14, 15). Rather than cylindrical dispensing tips situated inside of the spinneret, the top of the spinneret screws into the spinneret base, forming six rectangular orifices around the top perimeter of the spinneret, each measuring 6 × 0.15 mm (ID), where the PVA solution is extruded through under the influence of centrifugal force.

### PVA fibers

PVA fibers were produced from high-molecular-weight (HMW, 85k–124k Mw; 87–89% hydrolyzed) and low-molecular-weight (LMW, 30k–70k Mw, 87–90% hydrolyzed) PVA (Sigma Aldrich). Solutions were prepared using varying weight ratios of HMW PVA to LMW PVA dissolved to a total wt/vol of 10% in deionized (DI) water and held at 80°C for 5 days, with intermittent hand agitation to mix. Full dissolution is visually evident from the achievement of solution transparency. Viscosity for each spinning solution is given in Table 1 at 1,000 s⁻¹, as spinning operations will induce the shear-thinning regime for the solutions. Parameters for fiber production, including needle gauge size, rotation rates, and collection times, are also detailed in Table 1. PVA fibers were also produced using a modified Carnival King cotton candy machine to assess scalability and reduced manufacturing costs associated with basic technology (14, 15).

**TABLE 1:** Forcespinning conditions

Fiber	Solution	Viscosity (mPas)	Rotation rate (rpm)	Gauge size	Collection time (min)
PVA (10:0)-3000-30-7	PVA (10:0)	435 ± 5	3,000	30 (0.16 mm ID)	7
Cotton candy machine PVA (10:0)-3500-30-7	PVA (10:0)	435 ± 5	3,500	6 mm × 0.15 mm rectangular orifice	>10
PVA (9:1)-4000-32-4	PVA (9:1)	350 ± 10	4,000	32 (0.11 mm ID)	4
PVA (8:2)-4000-32-2	PVA (8:2)	288 ± 8	4,000	32 (0.11 mm ID)	2 to 3

### Fiber characterization

Fiber diameters and structural properties were characterized using scanning electron microscopy (SEM), magnifying them 100 and 1,000 times for comparison. The viscosity of each PVA solution was measured using a Haake MARS II rheometer. Surface area analyses were performed using Brunauer–Emmett–Teller (BET) gas sorption on a Micrometrics ASAP 2020 Surface Area and Porosity Analyzer (16). In preparing samples for surface area measurements, the sample tubes were evacuated to a pressure of 10 μm Hg (1.3 Pa) and then heated at a rate of 2°C min⁻¹ to a final temperature of 30°C. The samples were held at this temperature for 4 h under vacuum to ensure complete outgassing prior to analysis. Subsequent physisorption analysis was carried out at 77.35 K using ultra-high purity (UHP) nitrogen as the adsorbate. Surface areas were calculated by the Brunauer–Emmett–Teller (BET) method using five adsorption points in the relative pressure range (P/P₀) of 0.06–0.20. Thermal stability was assessed via thermogravimetric analysis (TGA) by ramping the temperature at 5°C/min to 300°C, followed by holding at 300°C for 1 h (17). All fibers were tested for water solubility in both phosphate-buffered saline (PBS) and nutrient broth growth media.

### Swab materials

Commercially sterilized swabs made from cotton (Pur-Wraps Swab, Puritan Medical Products, cat. no. 25-806 2PC), rayon (Puritan Swab, Puritan Medical Products, cat. no. 25-806 1WR), and macrofoam (Puritan, cat. no. 25-1607 1PF SC) were used for comparison with the PVA swabs. While CDC protocols recommend prewetting macrofoam swabs with PBS plus 0.04% Tween-80 (pH 7.2) or neutralizing buffer, PVA swab fibers dissolved in such solutions. To avoid premature dissolution of the PVA swabs while prewetting, the prewetting solution was standardized across all swab types to 10× PBS (6, 18). Pre-sterilized negative control swabs of each type were tested directly from packaging.

### Bacterial spores

*Bacillus thuringiensis* (Al Hakam) spores, collected in Iraq by the United Nations Special Commission, held in collection, and provided by Sandia National Laboratories, were prepared in-house following established sporulation and purification procedures similar to those described by Buhr et al. (2012) and Buhr et al. (2016), produced in sporulation medium over 3 days at 36°C, shaking at 250 RPM (19–21). Spores were harvested by centrifugation (5,000 × *g*, 10 min) and washed with 1× PBS twice before being treated with 1 mg/mL lysozyme and 0.01 mL/mL Tween 80 to ensure purity. Spore suspensions were diluted to concentrations of 1.3 × 10^5^ and 1.16 × 10^6^ colony-forming units (CFUs)/mL with ultrapure water and stored in ultrapure water or 95% ethanol (EtOH) at 4°C before testing. Spore preparations were confirmed to be >95% phase-bright spores by phase-contrast microscopy.

### Surface sampling and swab testing

Stainless steel coupons (10 × 10 cm) were cleaned and steam-sterilized, and 1 mL of 1.3 × 10^5^ CFU/mL bacterial spores, suspended in 95% ethanol (EtOH), was spread evenly over a 5.1 × 5.1 cm (2 × 2”) area on them using a sterile micropipette tip, placed in sterile and closed Petri dishes, and allowed to dry overnight inside a sanitized biosafety cabinet. Since PVA fibers partially dissolved when premoistened with neutralizing buffer (Hardy Diagnostics, cat. no. K105) but did not dissolve in sterile 10× PBS, 10× PBS was used to premoisten the PVA swab fibers in lieu of neutralizing buffer. Premoistened swabs were swiped across the 100 cm^2^ sample surface in an “S” pattern, horizontally, vertically, and then diagonally, rotating the swab at each interval so that a fresh side of the swab was being used for each direction, according to CDC protocol for surface sampling (3). All surface sampling experiments were performed in triplicate (*n* = 3) for each swab type and condition. Each replicate consisted of a separately inoculated surface coupon and independently performed swabbing and recovery procedure. Colony-forming unit (CFU) counts were determined for each replicate, and results are reported as mean ± standard deviation.

To compare how well each swab type releases spores, 100 µL of 1.3 × 10^5^ CFU/mL spore suspension, in water, was dispensed into each swab type using a micropipette before the sample was extracted and enumerated. To extract the captured spores from the swab fibers, each swab was placed in 5 mL of sterile PBS and mixed at high speed via vortex in 10-second bursts for a total of 2 min (5). Recovery efficacy was determined by plating log dilutions of extracted samples onto 3M Petrifilm Aerobic Count Plates, which were incubated overnight at 37°C before CFUs were enumerated. In an effort to prevent variation in technique, the same laboratorian prepped and extracted swab replicates.

### Data analysis

Spore recovery efficiency was calculated by comparing the CFUs collected and recovered from each swab type to the known total number of spores initially deposited onto the surface. CFUs were enumerated and analyzed to assess differences in performance among the tested swab materials.

## RESULTS

### PVA fiber production and characterization

SEM was used to measure the diameters of swab fibers, which were then averaged and analyzed via one-way ANOVA. Notably, the range and standard deviation of the measured rayon fibers (*n* = 4) were much smaller and more uniform than those of the cotton swab fibers (*n* = 8), with their average fiber diameter differing by around 5%, rayon being the smaller of the two. The PVA fiber compositions containing eight (*n* = 9) and nine parts (*n* = 4) HMW PVA produced fibers over 25% smaller in diameter than the fibers resulting from 100% HMW PVA (*n* = 8; *P* = 0.9977 and 0.9998, respectively). Interestingly, while the 10:0 HMW fibers forcespun by the cotton candy machine (*n* = 34) had a higher standard deviation than fibers of the same composition spun by the FibeRio Cyclone L-1000M Forcespinning system, the average fiber diameter was not significantly smaller (*P* ≤ 0.9999) (Table 2).

**TABLE 2:** Average diameters and diameter ranges of PVA and commercial fibers

Fiber	Avg. diameter (μm)	Range (μm)	Number of measurements	SD
Cotton	15.18	7.22–22.12	8	4.872
Rayon	13.15	12.37–14.43	4	0.95
PVA (10:0)-3000-30-7	0.85	0.58–1.13	8	0.20
PVA (9:1)-4000-32-4	0.65	0.48–0.81	4	0.14
PVA (8:2)4000-32-2	0.59	0.28–0.84	9	0.19
PVA (10:0) cotton candy machine	0.82	0.407–1.53	34	0.29

**Fig 1:**
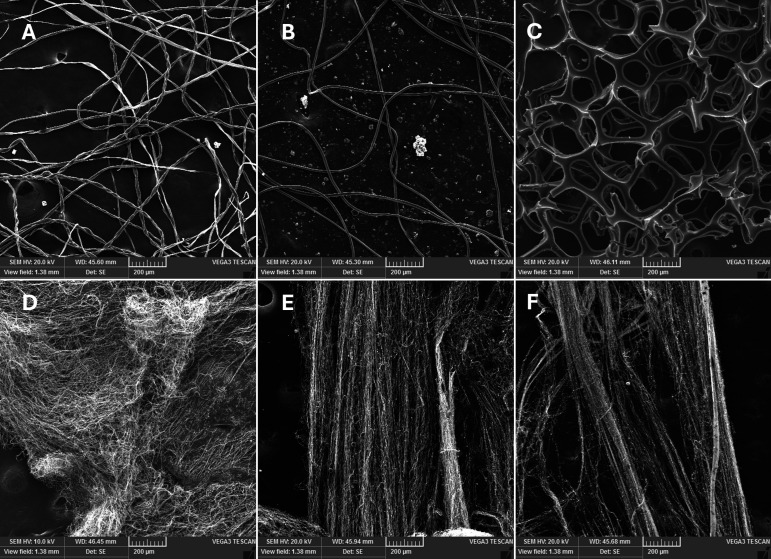
100× magnification SEM images of (**A**) cotton swab fibers, (**B**) rayon swab fibers, (**C**) macrofoam swab material, (**D**) PVA (10:0)-3000-30-7 swab fibers, (**E**) PVA (9:1)-4000-32-4 swab fibers, and (**F**) PVA (8:2)4000-32-2 swab fibers.

**Fig 2:**
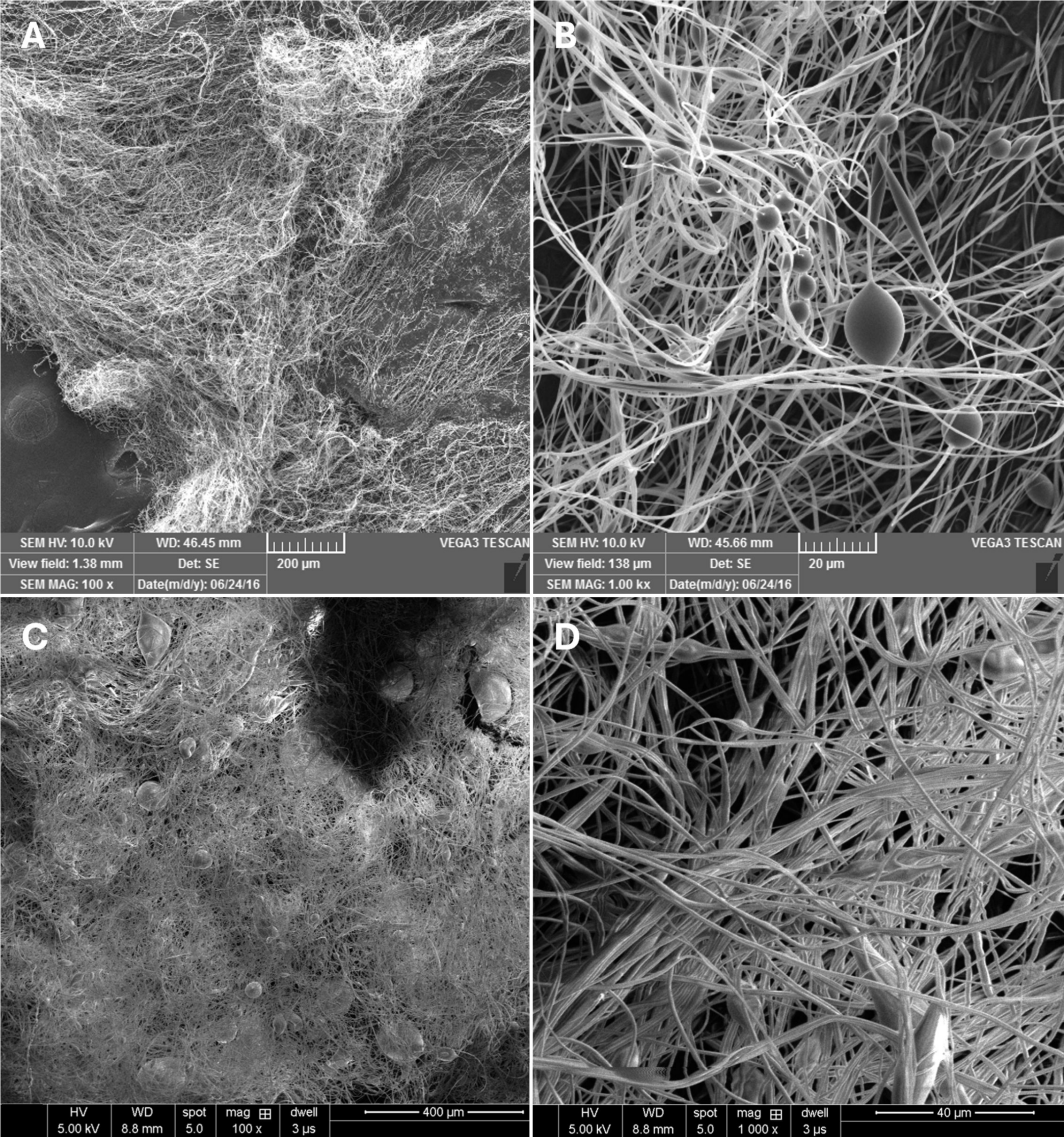
SEM images of 100% HMW PVA fibers forcespun using commercial-grade Forcespinning system: (**A**) 100× and (**B**) 1,000×. 100% HMW PVA fibers forcespun using cotton candy machine: (**C**) 100× and (**D**) 1,000×.

The forcespun PVA fibers produced using a modified FibeRio Cyclone L-1000M Forcespinning system displayed uniform diameters ranging from 0.5 to 1 µm, with occasional PVA bulb formations measuring up to 10 µm in diameter and 20 µm in length (Fig. 2). When comparing the PVA fiber characteristics to the other swab types, PVA (8:2)-4000-32-4 fibers had the smallest average diameter (0.59 µm), followed by PVA (9:1)-4000-32-4 (0.61 µm) and PVA (10:0)-3000-30-7 (0.86 µm) (Table 2). This is in contrast to the much larger average fiber diameters of cotton (15.18 µm) and rayon (13.15 µm). PVA fiber continuity, consistency, and diameter were further confirmed through scanning electron microscopy (SEM) and compared to the fiber structures of the comparative commercial swabs (Fig. 1).

To assess scalability, PVA fibers were also produced using a Carnival King cotton candy machine modified so that the spindle temperature is adjustable and the fiber collection platform is shaped to allow forcespun fibers to dry as they are collected (14, 15). PVA fibers generated using the cotton candy machine exhibited average diameters (0.82 µm), which are comparable to those produced by the FibeRio Cyclone L-1000M Forcespinning system. Figures 1 and 2 visually compare the same SEM image of PVA fibers forcespun using the commercial-grade Forcespinning system to the comparative swab materials (Fig. 1) and to the same PVA composition that was forcespun using a cotton candy machine (Fig. 2) (0.85 µm; *P* ≥ 0.9999) (Fig. 2). It is also important to note that cotton and rayon fiber diameters are both significantly larger than those of all other PVA-based fibers (*P* ≤ 0.0001) (Fig. 2). Because foam swabs are not composed of fibers, they were not directly compared to the fibrous swabs.

Interestingly, cotton swabs were shown to have roughly 13% more surface area (0.9098 m^2^/g) than the macrofoam swabs (0.7875 m^2^/g) and 76% more than the rayon (0.2156 m^2^/g), while 100% HMW PVA (2.6769 m^2^/g) was shown to have over twelve times the surface area of rayon and nearly three times that of cotton (see Table S1).

### Material characterization

#### Rheological properties of PVA in solution

The viscosity profiles of polyvinyl alcohol (PVA) solutions prior to forcespinning were characterized using a Haake MARS II rheometer at 25°C. The PVA (10:0)-3000-30-7 formulation, comprising 100% high-molecular-weight (HMW) PVA, exhibited non-Newtonian, shear-thinning behavior typical of concentrated polymer systems (Fig. S7 to S11). At low shear rates (0.1 s⁻¹), the solution displayed a viscosity of approximately 500 mPa·s, which decreased to around 430 mPa·s at higher shear rates (1000 s⁻¹). PVA (10:0)-3000-30-7, however, consistently maintained higher pre-forcespinning viscosity values than that of mixed molecular weight across the range of shear rates tested.

Dissolution testing of our PVA swabs shows that their fibers dissolve completely in 10 mL of water within 45–120 s at RT. Their thermal stability, as determined by thermogravimetric analysis (TGA), showed no detectable degradation of the fibers up to 250°C (see Supplementary material).

### Swab performance and spore recovery

**Fig 3:**
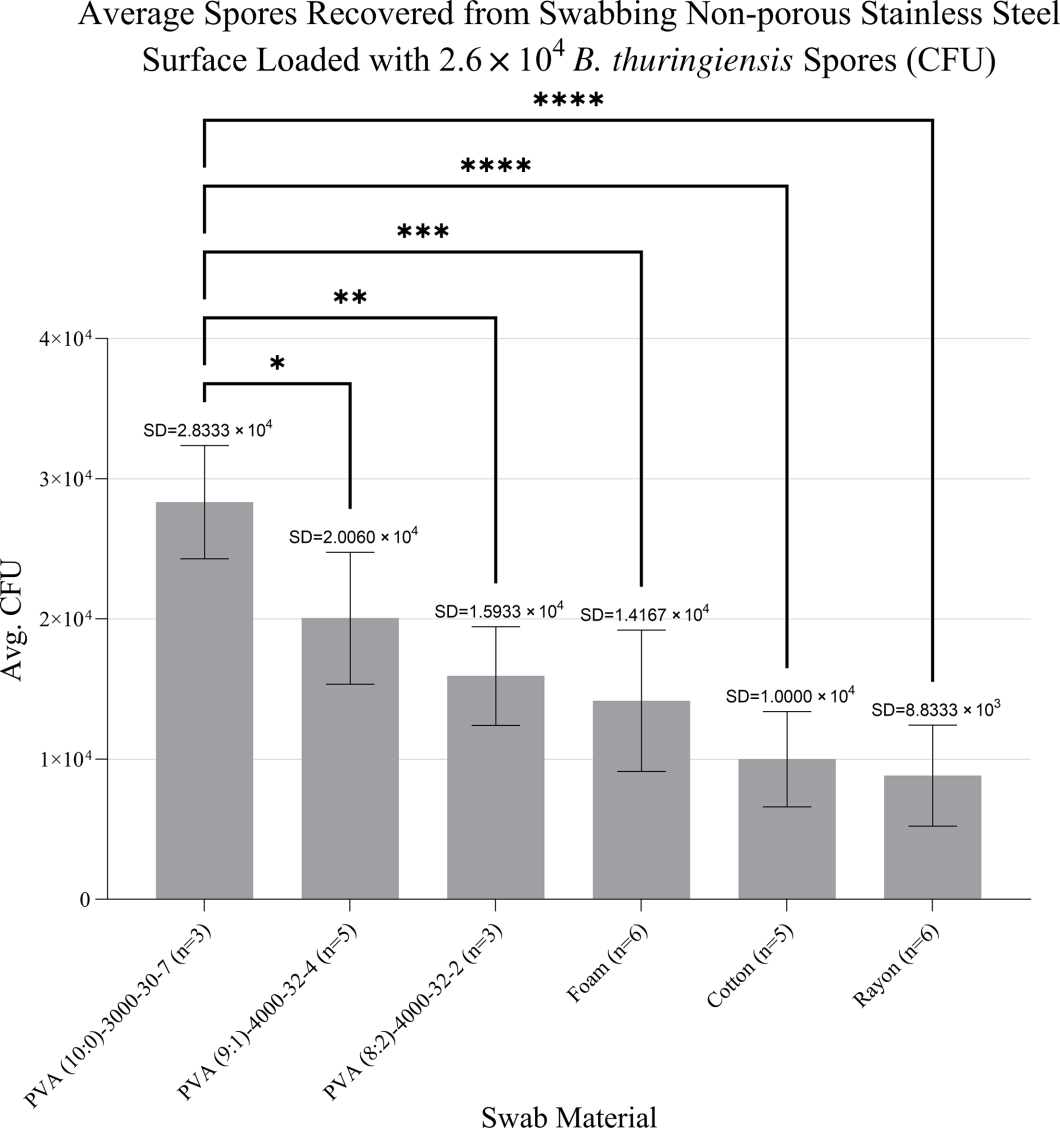
PVA recovers spores from non-porous surfaces significantly better than other swabs. Annotated error bars indicate standard deviation between replicated samples (* = *P* < 0.05, ** = *P* < 0.01, *** = *P* < 0.001, and **** = *P* < 0.0001).

The efficacy of the swabs forcespun from PVA in capturing and releasing *Bacillus thuringiensis* spores from a stainless-steel surface was evaluated and compared to cotton, rayon, and macrofoam swab materials. While the standard deviations (SD) of the three compositions of PVA swabs overlap with each other, the SD of fibers made from 100% HMW PVA does not overlap with those of the comparative swabs. Indeed, ANOVA analysis (α = 0.05) of the spore recovery of swabs made from 100% HMW PVA compared with those made of foam (*P* = 0.0004), cotton (*P* ≤ 0.0001), and rayon swabs (*P* ≤ 0.0001) shows a significant difference in recovery of spores deposited and dried onto a stainless-steel surface (Fig. 3).

**Fig 4:**
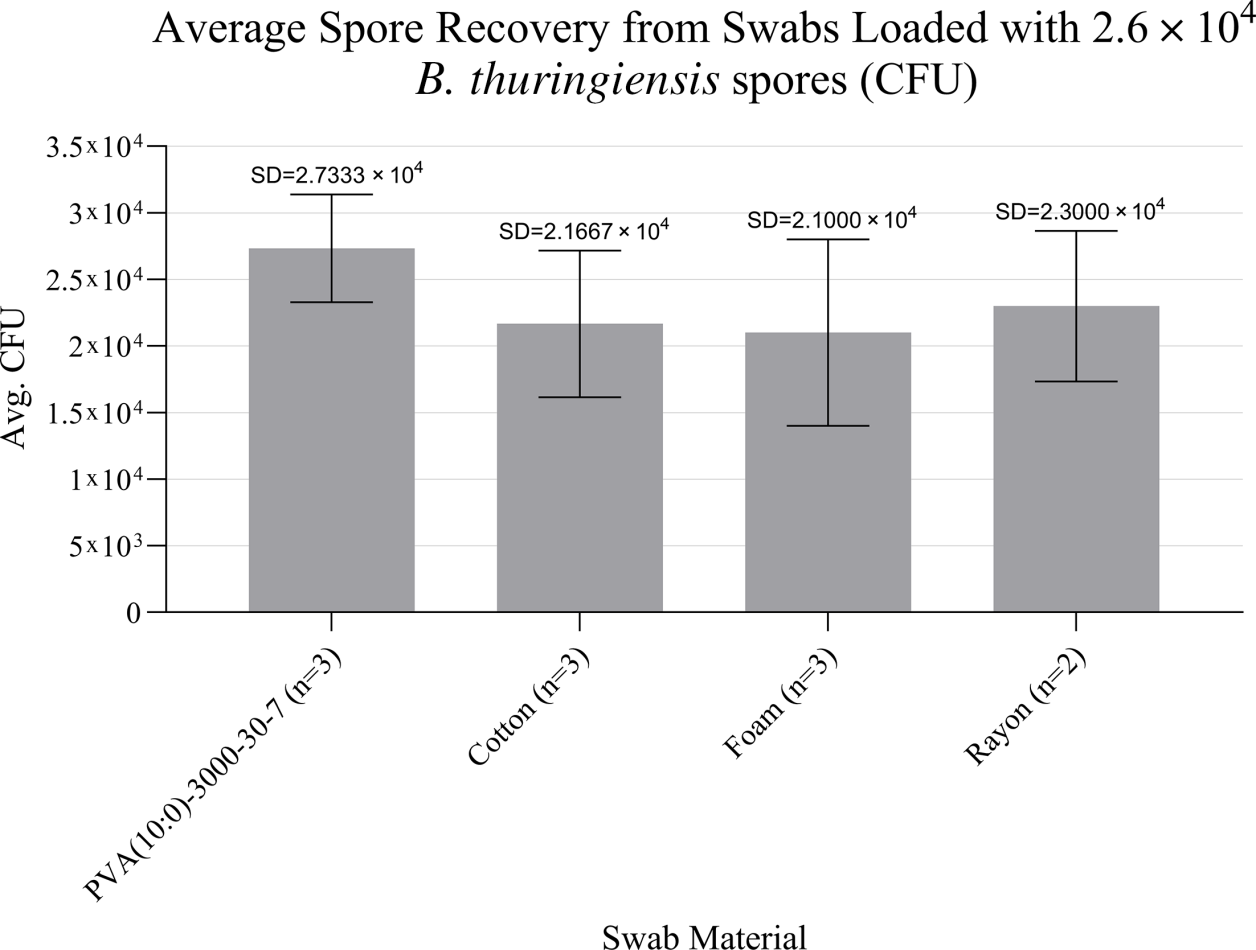
No significant difference was seen among spore recovery from swab types, each loaded with 2.6 × 10^4^ CFU/mL (swabs extracted into 5 mL extraction buffer) (error bars indicate standard deviation).

While examining the mechanical release of spores from swabs after vortex mixing, no significant difference was observed when compared to foam, cotton, or rayon swabs (*P* = 0.4375, 0.5162, and 0.7507, respectively), although PVA fibers showed comparably high extraction efficiency, releasing all captured material by completely dissolving into the extraction buffer (Fig. 4).

### Compatibility with bacterial growth

**Fig 5:**
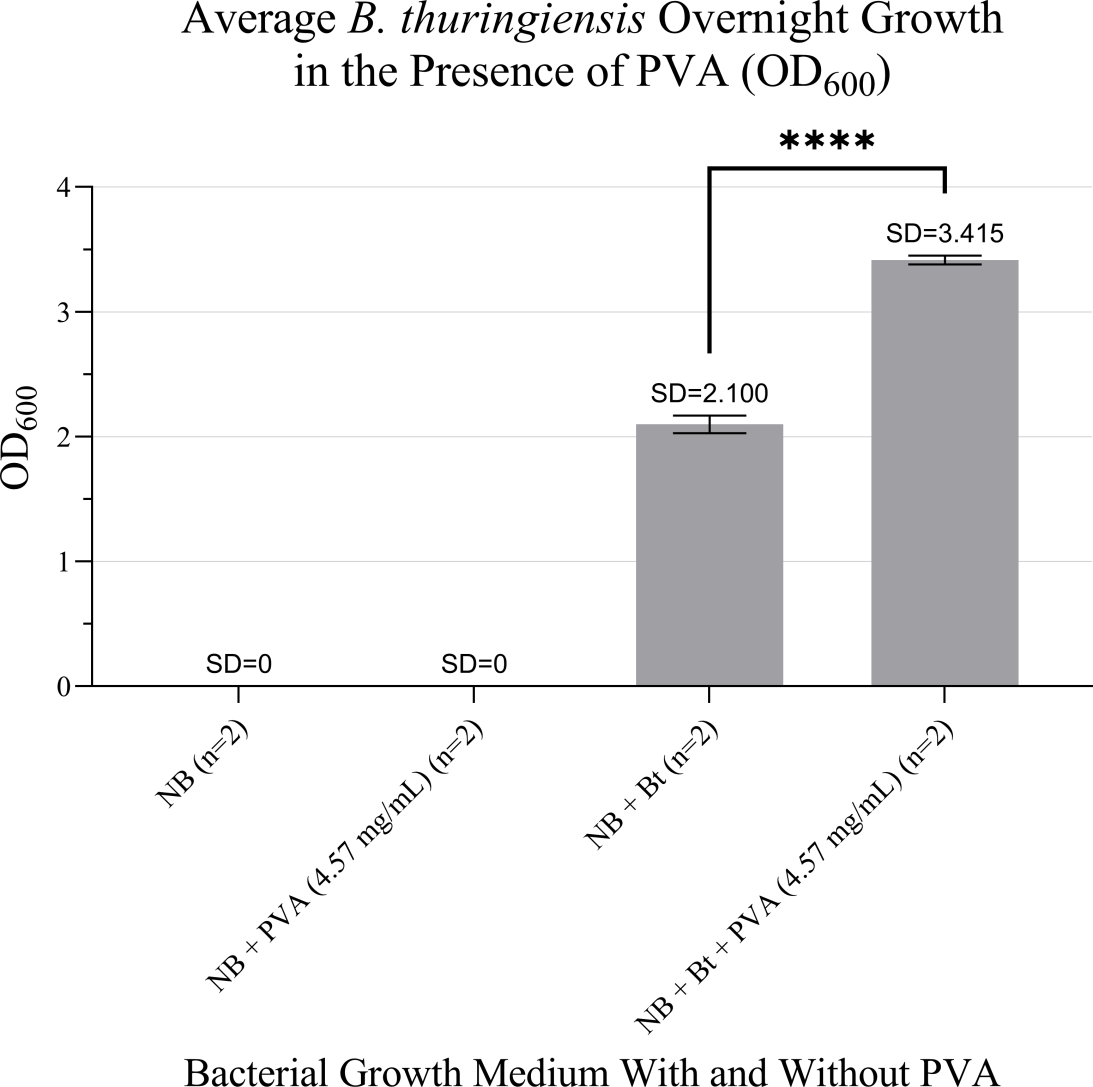
*Bacillus thuringiensis* growth is not inhibited by the presence of HM PVA in solution. Error bars depict standard deviation across replicates (**** = *P* ≤ 0.0001).

PVA fibers were also tested for their potential interference with spore germination and bacterial growth while in solution. Spores cultured in nutrient broth (NB) with dissolved 100% HMW PVA swab fibers exhibited similar growth to those cultured in nutrient broth without PVA (*P* ≥ 0.9999). This indicates that HMW PVA does not inhibit bacterial growth of *Bacillus thuringiensis*. While optical density (OD_600_) was not used to predict or measure bacterial growth in earlier testing here, this method indicates an approximate 53.2% increase in bacterial growth in the presence of dissolved PVA. These data suggest that HMW PVA does not adversely affect the viability of *Bacillus thuringiensis* (Fig. 5).

## DISCUSSION

This study demonstrates the successful development of water-soluble PVA swabs, which offer significant improvements in the efficiency of surface sampling for biological threats. Current surface sampling protocols rely on materials that are not water-soluble, which have been reported to release less than 40% of collected biological material and require more human intervention for analysis (5). The PVA swabs developed in this study dissolve completely in aqueous solutions, releasing 100% of the captured sample and can be more readily incorporated into automated analysis systems. This advance has the potential to significantly improve environmental sampling by increasing sensitivity in spore recovery, reducing sample size requirements, and decreasing turnaround time for contamination assessments (22).

### Swab size and surface area

We have shown that PVA swabs have an order of magnitude more surface area (2.6769 m^2^/g) than macrofoam (0.7875 m2/g), rayon (0.2156 m^2^/g), and cotton (0.9098 m^2^/g) swabs. This is likely due to their average fiber diameter (0.82–0.85 µm) being much smaller than those of cotton (15.18 µm) or rayon (13.15 µm), along with their superior uniformity and continuity within the individual fibers themselves. We have also shown that PVA fibers can be forcespun using an inexpensive cotton candy machine, demonstrating scalability without appreciably affecting the fiber diameter or the surface area of the resulting swab.

### No bacterial growth inhibition

While Phattarateera et al. suggest that hydroxyl radicals generated from PVA interacting with water may disrupt bacterial membranes in *Escherichia coli* and *Staphylococcus aureus*, demonstrating a small degree of antibacterial behavior, PVA is widely regarded as biocompatible and non-toxic (23). Indeed, it has been demonstrated that unmodified PVA films and hydrogels generally show no intrinsic antibacterial activity against *Escherichia coli*, *Staphylococcus aureus*, or *Bacillus subtilis*, with antimicrobial effects arising only after incorporation of active agents such metal oxide nanoparticles or surface functionalization (24–29). Supporting these reports, our results also indicate that water-soluble PVA swabs provide superior *Bacillus thuringiensis* spore recovery and release from non-porous surfaces compared to conventional swab materials, with no adverse effects on spore germination or bacterial growth observed.

### PVA swab performance

While forcespun mixtures of 10 and 20% LMW to HMW PVA were tested for differences in non-porous surface swabbing efficacy, fibers forcespun from 100% HMW PVA fibers demonstrated superior performance when compared to PVA fibers containing LMW PVA. The 100% HMW PVA also dramatically outperformed the commercially available swabs to which it was compared, as well as those reported by Panpradist et al. (4). Swabs produced by Forcespinning PVA fibers with varying ratios of high- and low-molecular-weight polymers recovered between 50% and 100% of deposited spores. In comparison, cotton, macrofoam, and rayon swabs recovered 19–54%, 23–77%, and 15–54% of deposited spores, respectively. These results indicate that even the lowest-performing PVA swabs outperformed all conventional swab types tested. The highest recovery was observed with swabs composed of 100% high-molecular-weight PVA, which recovered all deposited spores in every replicate (Fig. 3). Moreover, the scalability of the forcespun PVA fibers was further confirmed by successfully producing similar fibers using a modified cotton candy machine. The ability to produce high-quality fibers with affordable, non-specialized equipment suggests that this technology could be implemented in large-scale production, making it accessible for broader environmental and industrial applications (30).

### Material characterization and stability

PVA (10:0)-3000-30-7 (100% HMW PVA) consistently maintained higher viscosity values before forcespinning than those of mixed molecular weight across the range of shear rates tested. This elevated viscosity is attributed to the absence of low-molecular-weight (LMW) PVA, resulting in greater entanglement of polymer chains and increased flow resistance. Comparing single molecular weight systems showed the impact of molecular composition on solution rheology. Specifically, the 100% HMW PVA solution exhibited approximately 20% higher viscosity at low shear rates (0.1–1.0 s⁻¹) than the 90% HMW PVA and 80% HMW PVA formulations. This viscosity advantage persisted across the transition region, indicating that even a small substitution of HMW PVA with LMW PVA considerably influences rheological behavior. This data suggests that molecular weight distribution can be used to tune the properties of the forcespun fibers, which can be advantageous since the higher viscosity solutions, containing a greater proportion of HMW PVA, likely contribute to the structural integrity of the forcespun fibers (30). Beyond rheology, several characterizations confirmed that PVA fibers had suitability for diverse applications. Dissolution testing showed that fibers deposited onto swabs dissolved completely in 10 mL of water within 45–120 s at RT. The incorporation of diverse crosslinking agents, such as specific boron compounds, offers the potential to tailor dissolution rates to meet end-use requirements.

### Thermal stability

Thermogravimetric analysis showed that the PVA fibers are thermally stable up to 250°C, indicating that the fibers can withstand various environmental conditions during production and storage (see Supplementary material). Surface area analysis showed that PVA fibers possess up to three times the surface area of cotton fibers, which is likely to contribute to their superior spore capture efficiency (see Supplementary material). Additionally, X-ray diffraction analysis revealed that the PVA fibers are primarily amorphous, with some crystalline regions, which may influence their dissolution behavior and mechanical properties (30) (see Supplementary material). The rapid dissolution of PVA swabs in water further supports their application in sampling protocols, where complete and efficient sample recovery is essential.

### Applications and implications

The water-soluble nature of PVA swabs opens new possibilities for integrating sampling with microfluidic and automated detection systems (31). Their compatibility with aqueous media ensures that they can be used in conjunction with downstream analyses, such as microbial culture or molecular detection assays, without introducing contaminants or inhibitory compounds. Importantly, the PVA swabs did not interfere with the germination or growth of bacterial spores, further confirming their suitability for microbiological applications (5).

The ability to tune the dissolution rate of PVA fibers through the introduction of crosslinking agents, such as boron, could allow for the customization of these swabs for different environmental conditions. For example, slower-dissolving swabs may be advantageous for sampling in humid or wet environments, where premature dissolution could compromise sample collection.

### Limitations and future directions

While the PVA swabs performed exceptionally well in controlled laboratory conditions, further testing is needed in real-world settings to confirm their effectiveness in various environmental contexts, such as outdoor sampling and use in industrial contamination assessments. Additionally, while the scalability of PVA fiber production was demonstrated with a cotton candy machine, optimizing production methods for industrial-scale manufacturing will be essential for widespread adoption (22, 30). Future studies should also explore the application of PVA fibers for the sampling of a broader range of biological agents, including viruses and vegetative bacteria, to confirm their versatility across different pathogen types. Investigating the potential of PVA materials to capture smaller particles, such as aerosols, could further expand their utility in biological threat detection (31).

### Conclusion

In conclusion, this study highlights the potential of water-soluble PVA swabs to revolutionize environmental sampling for biological threats. Their superior spore recovery efficiency, scalability, and compatibility with microbiological analysis methods position them as a promising tool for improving contamination detection and decontamination processes. Future work will focus on optimizing the fibers for large-scale production and expanding their use to diverse environmental and pathogen contexts (30, 31).

## SUPPLEMENTAL MATERIAL

Supplemental material aem.00030-26-s0001.docx Supplemental methods, Fig. S1 to S20, and Tables S1 to S6.

ASM does not own the copyrights to Supplemental Material that may be linked to, or accessed through, an article. The authors have granted ASM a non-exclusive, world-wide license to publish the Supplemental Material files. Please contact the corresponding author directly for reuse.
