# Supplementary material for: Water-soluble swab material for environmental sampling
Source: Appl Environ Microbiol. 2026 Apr 29;92(5):e00030-26. doi: 10.1128/aem.00030-26 (PMC13188908; doi:10.1128/aem.00030-26)
Supplement: Supplemental material — Supplemental methods, Fig. S1 to S20, and Tables S1 to S6. [file aem.00030-26-s0001.docx]

**Supplementary Material**

PVA fibers were forcespun using Modified Forcespinning Machines; a commercial Forcespinning system (FibeRio Cyclone L-1000M Forcespinning™ system) (Supplementary Figure 1) and a modified Carnival King Cotton Candy Machine Supplementary Figures 2-5).


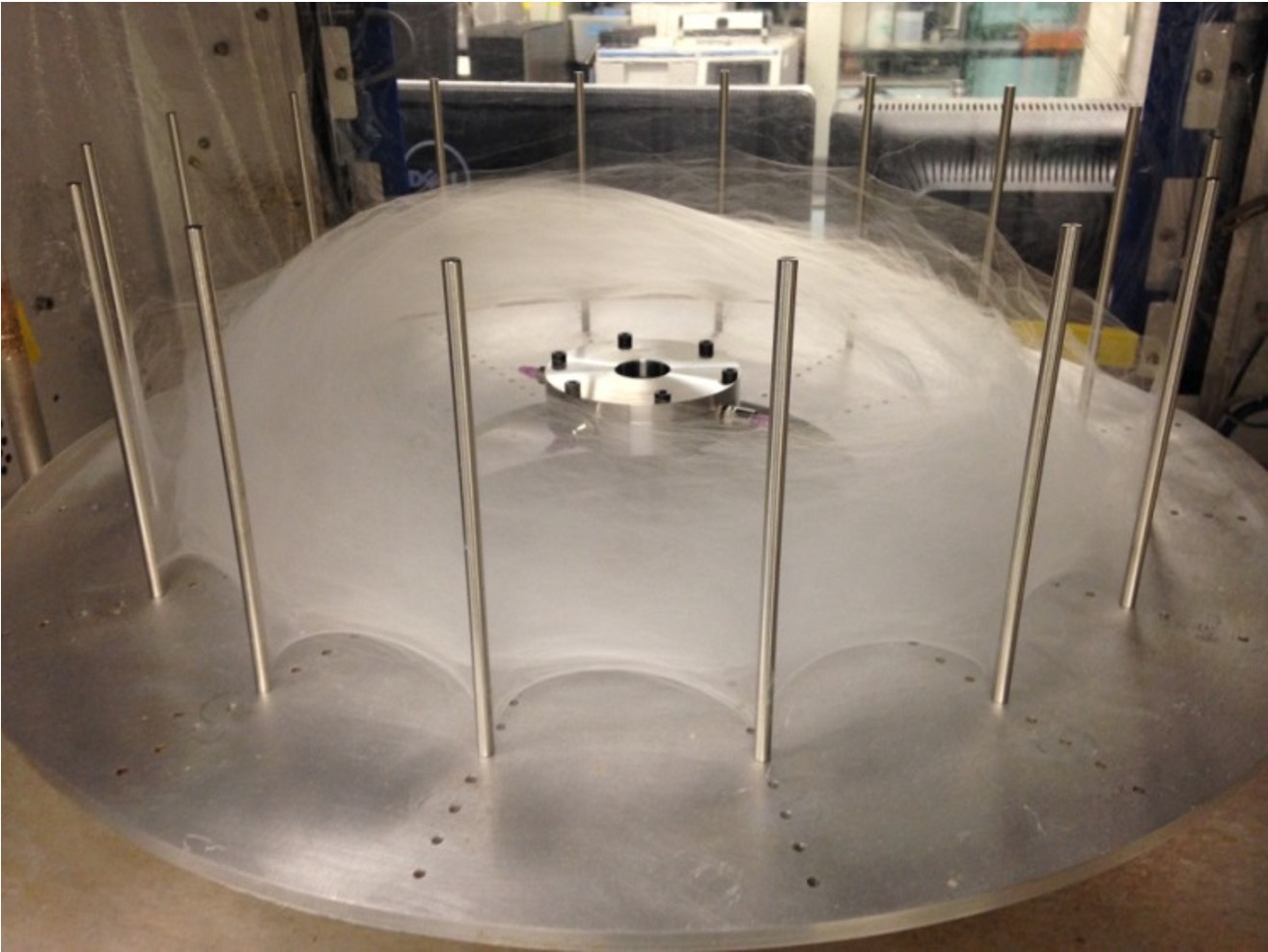


Supplementary Figure 1. Forcespinning™ 100% HMW PVA fibers


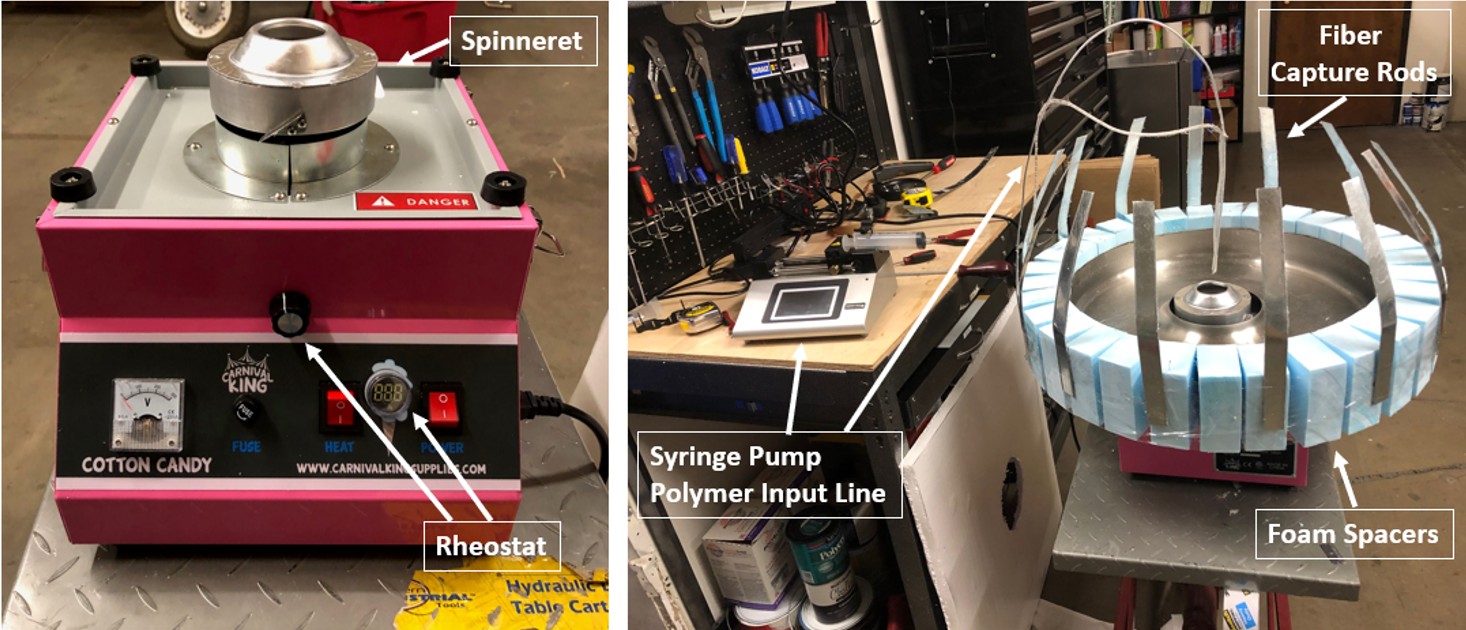


Supplementary Figure 2. Carnival King model 382CCME21 Cotton Candy Machine with added rheostat for spinneret temperature control. Fiber spinneret rotates at a non-adjustable 3500 RPM and is automatically heated to temperatures in excess of 210 °C, unadjusted. The added rheostat was made to hold the spinneret at a constant average temperature of 57-67 °C (SD=3.5)


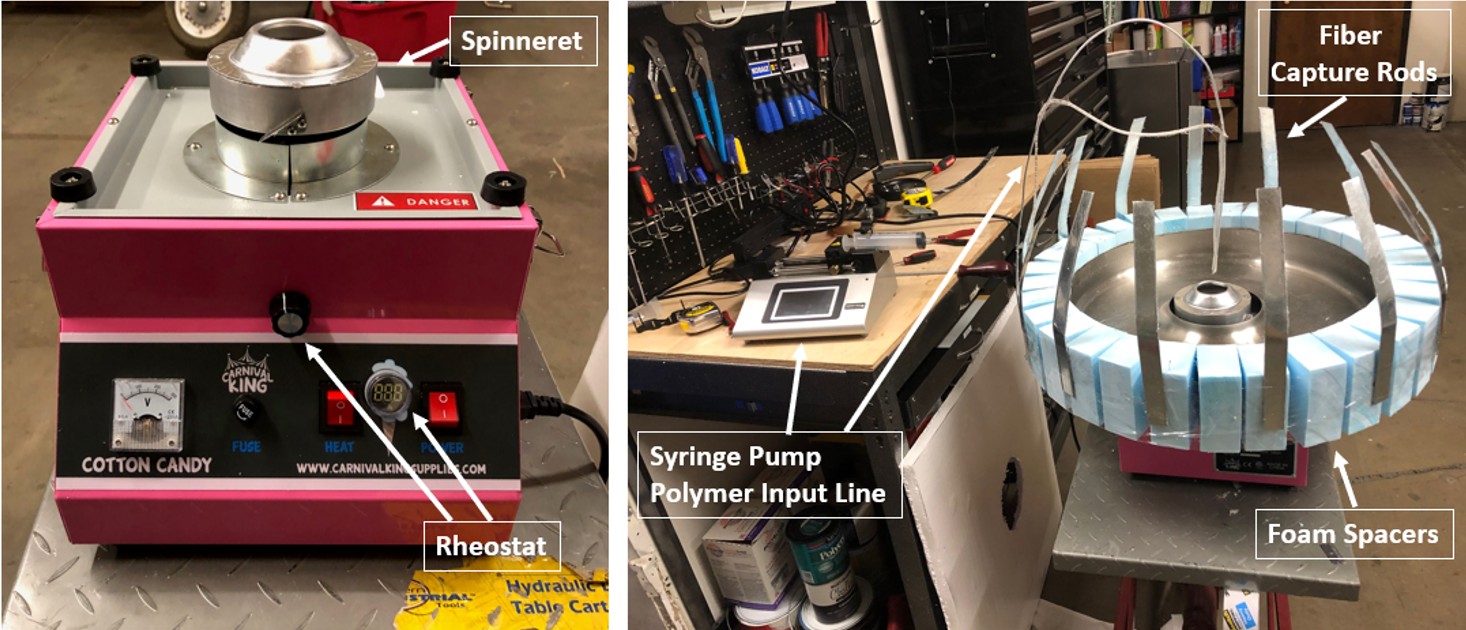


Supplementary Figure 3. Modified cotton candy machine

The cotton candy machine was modified to enable controlled polymer processing and improve fiber collection. A syringe pump was added to deliver polymer solution directly into the heated spinneret. To increase in-flight fiber drying time, the effective diameter of the collection bowl was expanded by attaching foam blocks around its perimeter where 1-inch-wide vertical collection slats were affixed to the exterior of them, extending the collection diameter from 19 5/8 inches to 25 3/4 inches. A wire support structure was installed to guide and stabilize the tubing connecting the syringe pump to the spinneret.


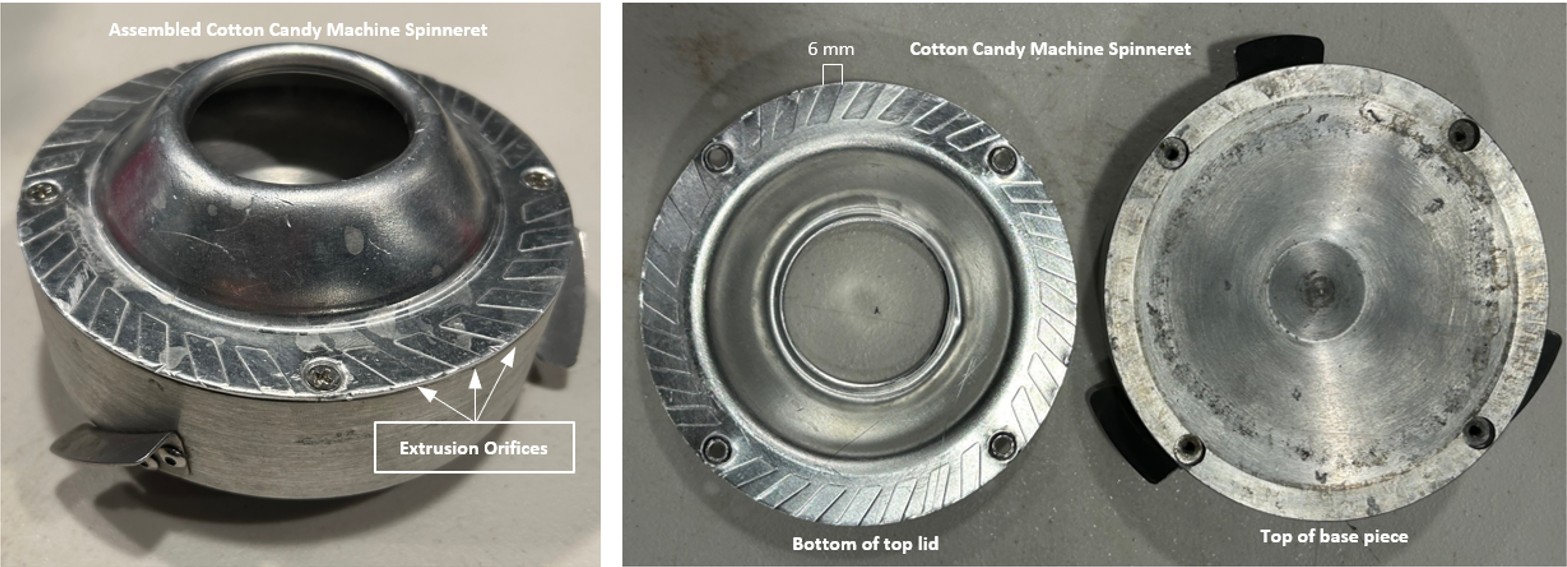


Supplementary Figure 4. Carnival King model 382CCME21 Cotton Candy Machine Two-piece Spinneret. The spinneret includes groves stamped into the top piece, measuring 6 mm wide and 0.15 mm deep and held flush against the flat bottom portion by four screws, producing a total of 28 rectangular orifices of the same size.


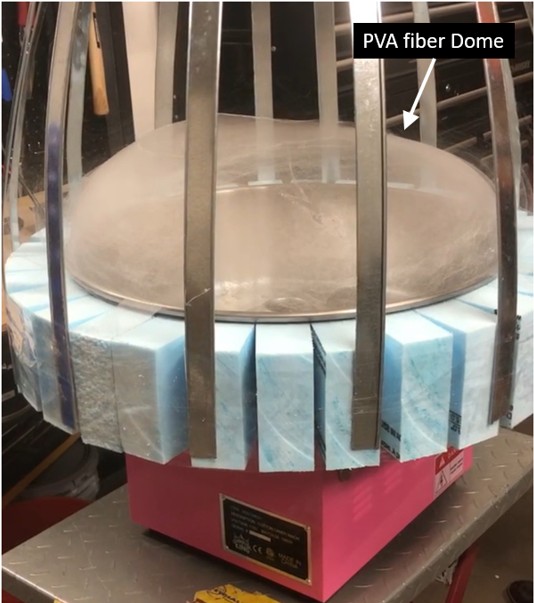


Supplementary Figure 5. PVA forcespun fiber dome created by a modified Carnival King model 382CCME21 cotton candy machine.

**Comparative swab materials**

Three commercially available swab materials were compared to fibers made from three PVA formulations. Each PVA swab consisted of approximately 40 mg of PVA fibers each that were held and immobilized by flame-sterilized metal tweezers during premoistening and swabbing. Negative control swabs were analyzed for the presence of *B. thuringiensis* straight from the packaging.


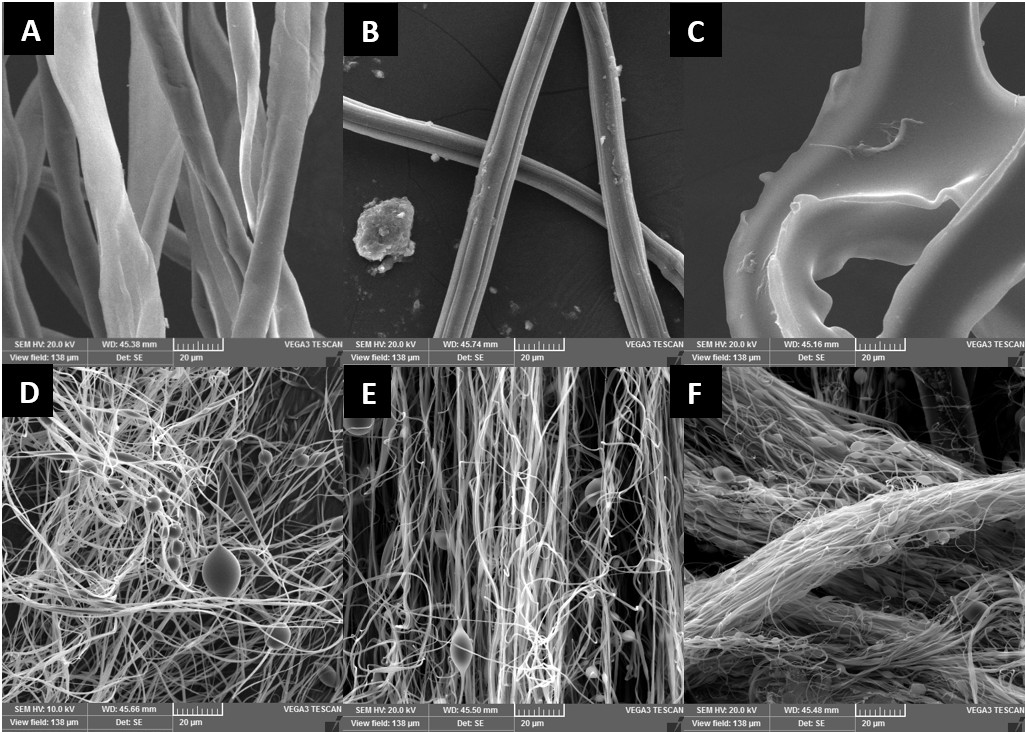
Supplementary Figure 6. 1000-times magnification SEM images of (A) cotton swab fibers, (B) Rayon swab fibers, (C) macrofoam swab material, (D) 100% HMW PVA fibers, (E) 90% HMW PVA fibers, and (F) 80% HMW PVA fibers

**PVA Materials Characterization**

**Viscosity**


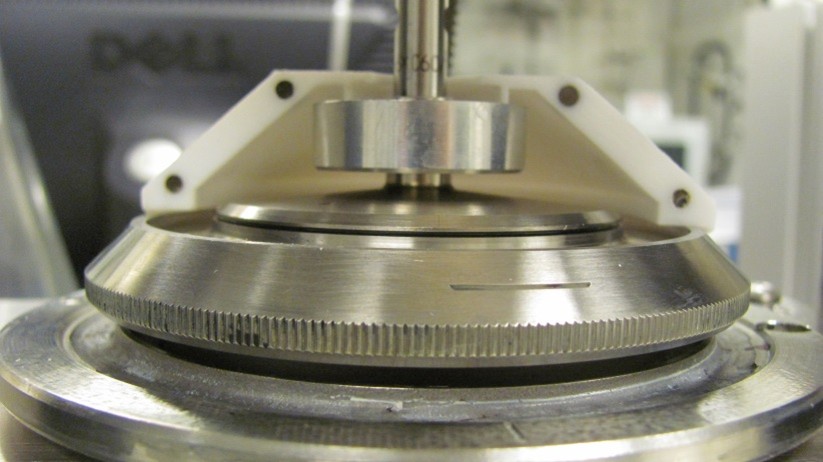


PTFE housing

Cup containing paraffin oil

Top rotating plate

Bottom stationary plate

Supplementary Figure 7. Haake MARS II rheometer set-up. ~1 mL of PVA solution was added between the two metal plates and were enclosed in a PTFE housing containing paraffin oil and a pipe cleaner ring soaked in DI water to help retain moisture.

Each measurement proceeded as follows:

1. Rotate at 1000 s^-1^ for 60 sec to remove air bubbles from the solution,
2. Rest for 60 s without rotation,
3. Rotation in logarithmic steps from a shear rate of 0.1 s^-1^ to 1000 s^-1^ to collect a total of 40 data points.

The gap was set to 0.052 mm, the thermal expansion coefficient was set at 1.100 μm/C, and the temperature of the bottom plate was kept at 25 °C via a Haake Phoenix C25P circulating water bath. Each 1 mL solution sample was measured twice with as little time delay between measurements as possible. All solutions were measured in triplicate, totaling six measurements per solution.

The viscosity of aqueous solutions of PVA used in Forcespinning™ comprised of different ratios of high and low molecular weights (MW) of PVA were tested. While PVA comprised of 90% high MW PVA and 10% low MW was shown to be at roughly one order of magnitude higher than that comprised of 80% high MW and 20% low MW PVA, the solution containing 0% low MW PVA was shown to be two orders of magnitude more viscus than that made with 90% high MW PVA and 10% low MW. The units of viscosity are shown in millipascal-second (mPa·s), shear stress in Pascal (Pa), and shear rate in s^-1^. The black line corresponds to the plot of shear stress vs. shear rate. The slope of this line gives the viscosity, shown in red, blue, or green.


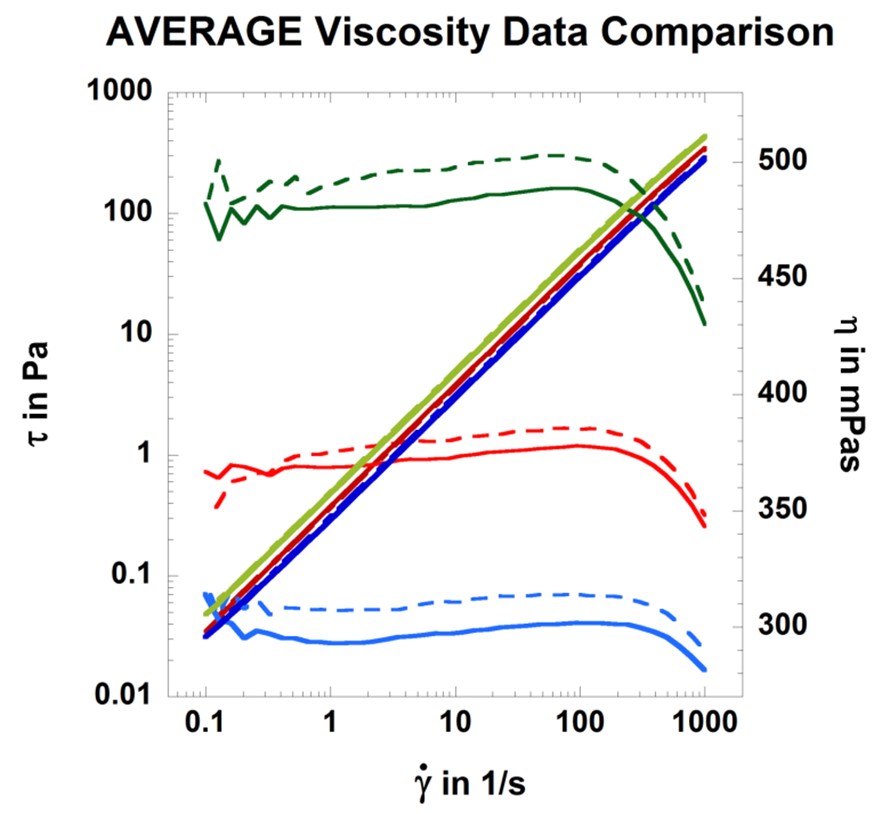


**PVA (10:0)**

**PVA (9:1)**

**PVA (8:2)**

Supplementary Figure 8. Average viscosity measurements for each PVA solution.

Raw and average viscosity data for PVA solutions before electrospinning which are comprised of 100% high molecular weight PVA, 90% high molecular weight and 10% low molecular weight, and 80% high molecular weight and 20% low molecular weight PVA.


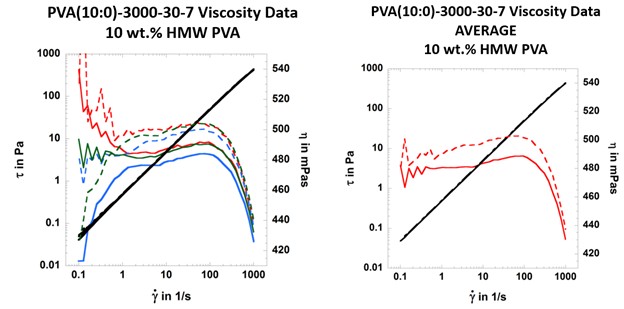


Supplementary Figure 9. Raw viscosity data for the PVA(10:0)-3000-30-7 solution (left) and average data (right). Different colors denote a different 1 mL sample of the same solution. The 2^nd^ run of each sample is denoted by dotted lines.


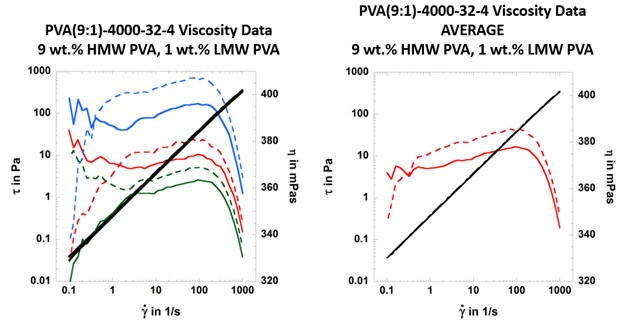


Supplementary Figure 10. Raw viscosity data for the PVA (9:1) solution (left) and average data (right). Different colors denote a different 1 mL sample of the same solution. The 2^nd^ run of each sample is denoted by dotted lines.


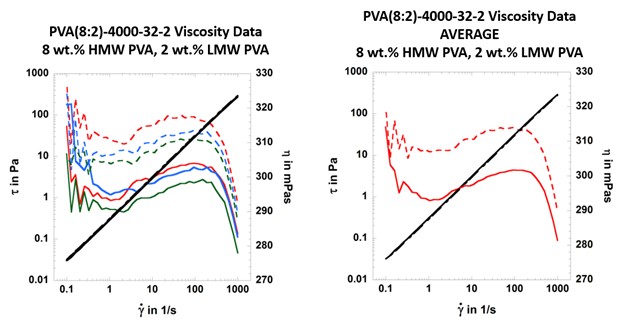


Supplementary Figure 11. Raw viscosity data for the PVA (8:2) solution (left) and average data (right). Different colors denote a different 1 mL sample of the same solution. The 2^nd^ run of each sample is denoted by dotted lines.

**PVA thermal stability**

The thermal stability of the PVA used in this study was measured via thermogravimetric analysis (TGA)^29^. TGA was performed on commercial PVA powders and forcespun PVA fibers in air by temperature ramping at 5 °C/min to 300 °C and then holding at 300 °C for 1 h. Samples were heated in platinum pans.


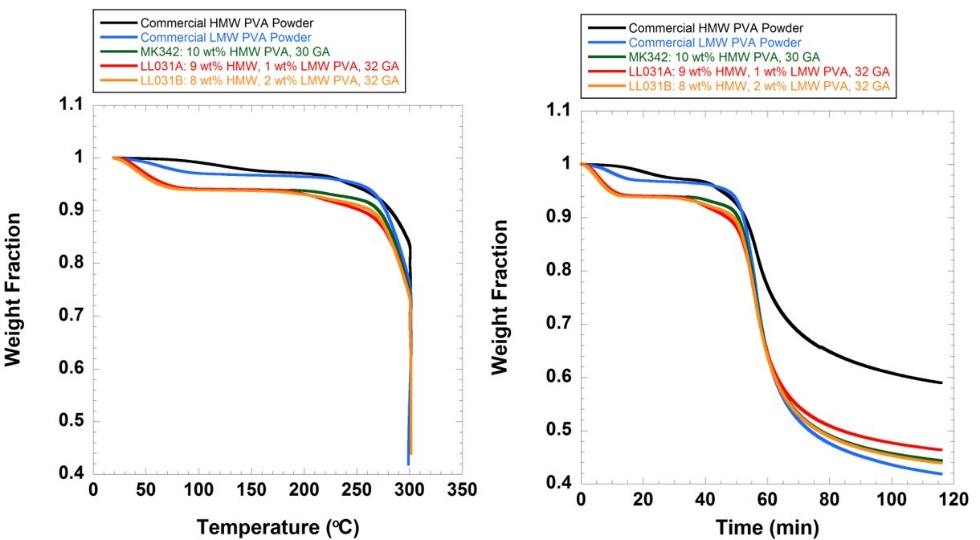


Supplementary Figure 12. Thermal stability of each PVA solution.

High molecular weight PVA, undiluted as well as diluted to 10%, 9%, and 8%, as well as low molecular weight PVA, show constant weight fraction retention up to temperatures exceeding 250 °C, up to about an hour. This shows that PVA in these forms is thermally stable and can withstand high temperatures during manufacturing without degradation and that the resulting fibers are not likely to degrade or break down in high environmental or storage temperatures.

**Surface Area Analysis - Brunauer-Emmett-Teller (BET)^28^**

Gas sorption experiments were performed on a Micrometrics ASAP 2020 Surface Area and Porosity Analyzer. Two different procedures were used to degas samples: Method 1: Evacuate sample tube to 10 μm Hg, ramp at 10 °C/min to 100 °C and hold at 100 °C for 20 hours. Method 2: Evacuate sample tube to 10 μm Hg, ramp at 2 °C/min to 30 °C and hold at 30 °C for 4 hours.

Analysis was carried out at 77.35 K using ultra high purity (UHP) nitrogen as the adsorbate. Surface areas were calculated by the BET method using five adsorption points in the range P/P0 = 0.06 to 0.2

Where degassing Method 1 resulted in PVA fibers turning slightly brown, degassing Method 2 did not. For this reason, degassing Method 2 was used for PVA fibers and degassing Method 1 was used for commercial swab materials.

After the sample was degassed through ramping the temperature 10 °C/min to 100 °C and hold at 100 °C for 20 hours the PVA fibers turned slightly brown as shown in Supplementary Figure 13, so the temperature ramping was changed to 2 °C/min to 30 °C and hold at 30 °C for 4 hours in order to preserve the fibers.


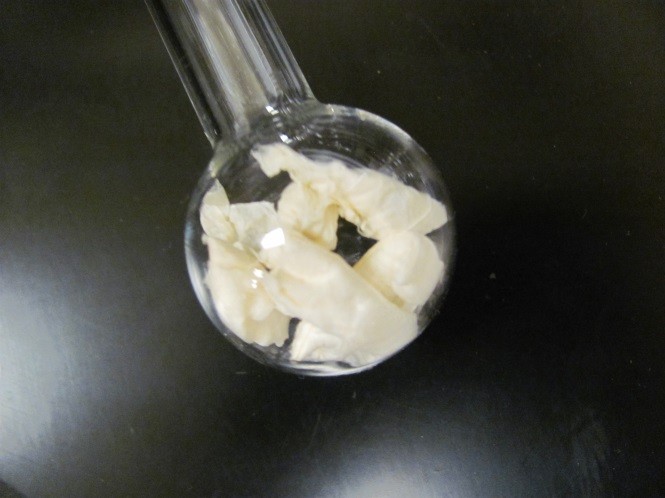


Supplementary Figure 13. The image above shows an PVA(10:0)-3000-30-7 sample after undergoing degas Method 1.

In comparing the surface area between commercially available swabs, cotton fibers were shown to have roughly 13% more surface area than macrofoam and 76% more than found in rayon fibers. The forcespun PVA fibers were shown to have up to 3 times the surface area of cotton fibers.

Supplementary Table 1: BET data comparing PVA to commercial off the shelf swabs. Grey highlighted data denotes samples degassed using Method 1.

| **Name** | **BET Surface Area (m2/g)** | **BJH adsorption pore volume (cm3/g)** | **BJH desorption pore volume**  **(cm3/g)** | **BJH Adsorption average pore width (Å)** | **BJH Desorption average pore width (Å)** |
| --- | --- | --- | --- | --- | --- |
| **PVA(10:0)-3000-30-7**  (10 wt% HMW PVA fibers) | 2.1520 | 0.003976 | 0.003463 | 69.3781 | 61.2645 |
| **PVA(10:0)-3000-30-7** (10 wt% HMW PVA fibers) | 2.6769 | 0.00526 | 0.004902 | 70.608 | 91.186 |
| **PVA(9:1)-4000-32-4** (9% HMW, 1% LMW PVA) | 1.3713 | 0.023593 | 0.023810 | 1091.359 | 902.390 |
| **PVA(9:1)-4000-32-4** (9% HMW, 1% LMW PVA) | 2.9932 | 0.005313 | 0.004398 | 61.902 | 120.868 |
| **PVA(8:2)-4000-32-2** (8% HMW, 2% LMW PVA) | 2.1291 | 0.013725 | 0.013940 | 335.502 | 552.941 |
| Foam  (Puritan 25-1607 1PF SC) | 0.7875 | 0.001503 | 0.000711 | 58.224 | 314.755 |
| Cotton  (Puritan 25-806 2PC) | 0.9098 | 0.016830 | 0.016814 | 667.231 | 705.166 |
| Rayon  (Puritan 25-806 1WR) | 0.2156 | 0.007900 | 0.007900 | 785.522 | 690.268 |

**X-Ray Diffraction (XRD)**

The crystal structure of the forcespun PVA fibers were analyzed via X-Ray Diffraction and found to have moderate crystalline structure rendering them amorphous in nature.


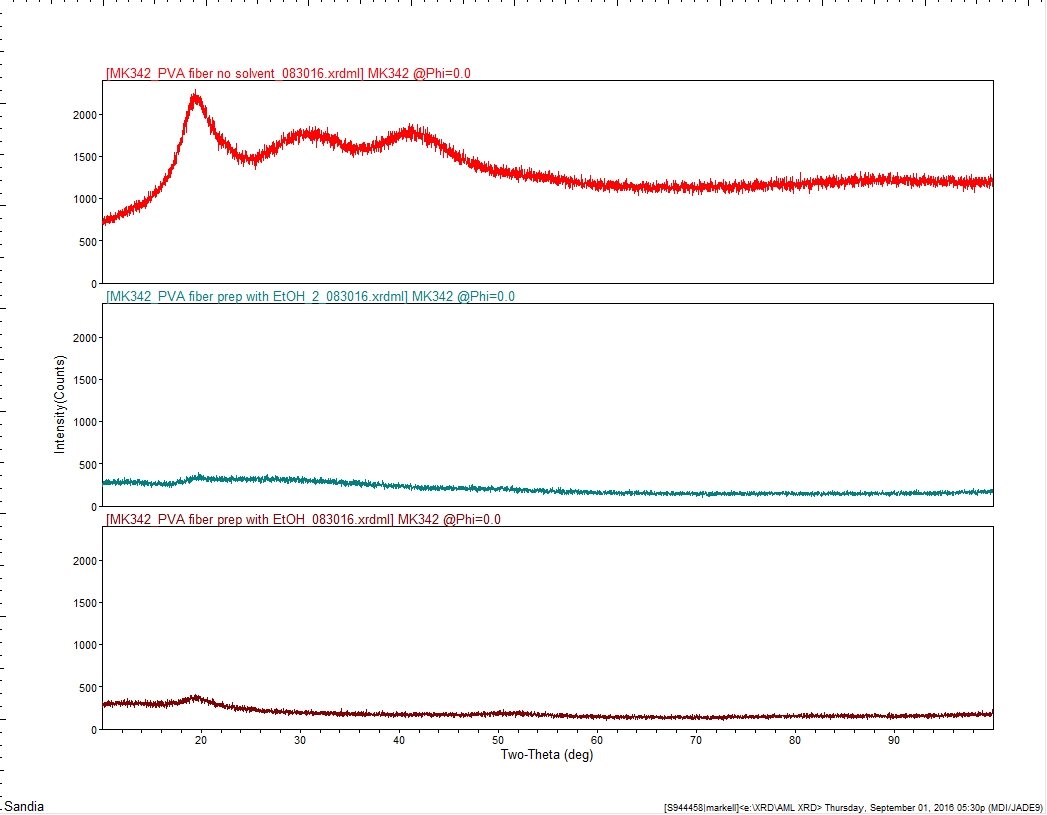


Supplementary Figure 14. Above: XRD spectra for PVA(10:0)-3000-30-7 fibers (10% HMW).

XRD samples were prepared using two different methods. Method 1: A piece of PVA fibers was cut into roughly the shape of the XRD sample holder. It was positioned in the sample holder so that it was as flat as possible and level with the plastic sides. Method 2: 2-3 drops of ethanol were dropped onto a no background silicon XRD sample holder. A small sheet of PVA fibers was then stretched over the ethanol as flat as possible. 2-3 more drops of ethanol were added on top of the fibers. The sample was allowed to air dry.

The XRD shows higher X-ray intensity peaks at lower angles and continuous intensity at 50 and higher angles. Peaks at angles 20, 30, and 42 indicate specific and well-defined crystallographic planes within the fiber structure resulting from our sample preparation method.

**PVA Fiber Solubility**

PVA fibers (40 mg) were shown to dissolve completely in 10 mL of DI water in minutes but dissolved incompletely in 10 mL of growth medium when mixed at room temperature (rt) for the same amount of time. PVA swabs did dissolve completely when incubated on an orbital shaker at 37 °C at 250 rpm for 60 minutes. This is still considered to be a fast dissolution time when compared to that of calcium alginate swabs which can take up to several weeks to fully dissolve. Introducing varying small amounts of a crosslinking agent such as boron into the PVA solution to be forcespun, may decrease the rate of fiber dissolution into water, thus creating a method to dictate how fast the resulting swab will dissolve based on the degree of polymer crosslinking in this way. Swabs with a slightly slower dissolution rate may be useful when swabbing wet or liquid environmental surfaces for samples.

**Sampling**

The swabbing method described in *Surface sampling procedures for Bacillus anthracis spores from smooth, non-porous surfaces*, set forth by the CDC, was used when swabbing stainless steel coupons^3^. Since PVA fibers partially dissolve when premoistened with Neutralizing buffer (Hardy Diagnostics, Cat# K105) and did not dissolve when 10X PBS was used, sterile 10X PBS was used to premoisten only the PVA swab fibers^5^. Premoistened swabs were swiped across the spore-laden coupons in a horizontal ‘S’ pattern, then vertically, and then diagonally, rotating the swab at each interval so that a fresh side of the swab was being used for each direction^3^. Each swab was then placed into a 15 mL vial containing 5 mL of sterile PBS (pH 7.2) with 0.04% Tween™ 80 and the swab handle aseptically broken off leaving the swab fibers immersed in the capped and sealed tube. All tubes containing swabs were mixed at high speed via vortex, in 10 s bursts, for 2 min to mechanically release spores from the swab fibers. All swabs of each material were incubated at 37 °C at 250 rpm in order to promote the dissolution of the PVA swabs while maintaining treatment consistency between all swab types. Each liquid sample was serially diluted, where needed, and plated on 3M™ Petrifilm™ Aerobic Count Plates to determine the spore collection and release efficacy of each swab type.

**Microscopy**

Spores were fixed in solution containing 4% Paraformaldehyde, 1% Glutaraldehyde, and 0.1M Millonig’s Phosphate Buffer (pH 7.4) and then imaged via Scanning Electron Microscopy (SEM).

**Data Analysis**

The spore stock concentration was determined by serially diluting and plating each dilution on 3M™ Petrifilm™ Aerobic Count Plates. Colony forming units (CFU’s) presented by each dilution, representing spores, were counted and the spore concentrations were calculated for the initial spore stocks. The resulting concentration was used to determine how many CFU’s were deposited onto a given substrate. The number of spores that were released from the swabs were enumerated in the same way and compared to the total number of CFU’s that were calculated to have been initially deposited.

Supplementary Table 5: PVA does not inhibit bacterial growth. Inoculated 10 mL NB and NB+PVA with 100μL of Bt spore stock (1.16x10^6^ CFU/mL). Incubated 16 hrs., 37°C, @ 250rpm. PVA(10:0)-3000-30-7 concentrations yielded by typical swab mass are shown not to inhibit bacterial growth (OD_600_).

|  |  |  | Sample 1 | Sample 2 | Sample 3 |  |
| --- | --- | --- | --- | --- | --- | --- |
| NB | | | 0 | 0 | 0 |  |
| NB + 4.57 mg/ml PVA | | | 0 | 0 | 0 |  |
| NB + 4.57 mg/ml PVA + Bt spores | | | 3.44 | 3.39 | 3.29 |  |
| NB + Bt spores | | | 2.15 | 2.05 | 2.41 |  |
|  |  |  |  |  |  |  |
